# Optical Electrophysiology in the Developing Heart

**DOI:** 10.3390/jcdd5020028

**Published:** 2018-05-11

**Authors:** Kandace Thomas, Julie Goudy, Trevor Henley, Michael Bressan

**Affiliations:** Department of Cell Biology and Physiology, McAllister Heart Institute, University of North Carolina at Chapel Hill, Chapel Hill, NC 27599, USA; kandacet@email.unc.edu (K.T.); julie_goudy@med.unc.edu (J.G.); thenley@med.unc.edu (T.H.)

**Keywords:** cardiac development, optical mapping, cardiac conduction system, physiological imaging

## Abstract

The heart is the first organ system to form in the embryo. Over the course of development, cardiomyocytes with differing morphogenetic, molecular, and physiological characteristics are specified and differentiate and integrate with one another to assemble a coordinated electromechanical pumping system that can function independently of any external stimulus. As congenital malformation of the heart presents the leading class of birth defects seen in humans, the molecular genetics of heart development have garnered much attention over the last half century. However, understanding how genetic perturbations manifest at the level of the individual cell function remains challenging to investigate. Some of the barriers that have limited our capacity to construct high-resolution, comprehensive models of cardiac physiological maturation are rapidly being removed by advancements in the reagents and instrumentation available for high-speed live imaging. In this review, we briefly introduce the history of imaging approaches for assessing cardiac development, describe some of the reagents and tools required to perform live imaging in the developing heart, and discuss how the combination of modern imaging modalities and physiological probes can be used to scale from subcellular to whole-organ analysis. Through these types of imaging approaches, critical insights into the processes of cardiac physiological development can be directly examined in real-time. Moving forward, the synthesis of modern molecular biology and imaging approaches will open novel avenues to investigate the mechanisms of cardiomyocyte maturation, providing insight into the etiology of congenital heart defects, as well as serving to direct approaches for designing stem-cell or regenerative medicine protocols for clinical application.

## 1. Introduction

The heart is an electrochemically oscillating muscular syncytium that generates the biomechanical forces required to pump fluid through the circulatory system. As for all muscle beds in the body, cardiac activation is stimulated by voltage-dependent changes in plasma membrane ion permeability. Large changes in the ion current flow trigger the self-sustaining electrical impulses, or action potentials, required for contraction. Electrical propagation in the heart is highly regulated by a specialized subpopulation of cells referred to as the cardiac conduction system (CCS). The CCS is completely contained within the heart and can rhythmically initiate and coordinate the impulse propagation required for cardiac function independent of any external stimuli.

**Figure 1:**
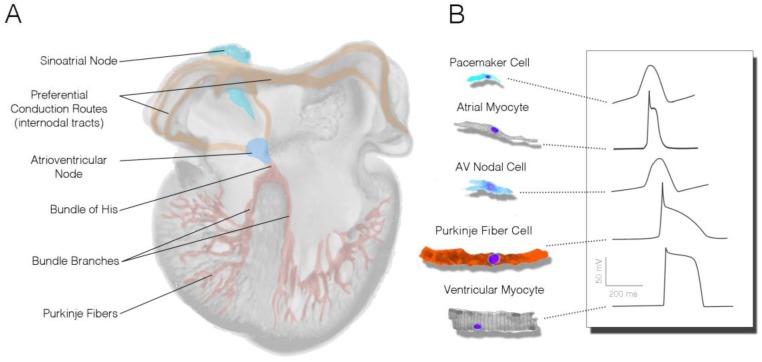
Organization of the mature cardiac conduction system. (**A**) Schematic of a four-chambered heart indicating the relative positions of the specialized subcomponents of the cardiac conduction system; (**B**) Relative cytoarchitecture and action potential characteristics of cells from various conduction system and working myocardial populations.

In the mature heart, the CCS is comprised of the sinoatrial node (SAN), the atrioventricular node (AVN), the bundle of His, bundle branches and Purkinje fibers (Figure 1A). The electrical impulses that induce contraction are initiated by pacemaker cells within the SAN and are then rapidly conducted across the atrial myocardium. Propagation is then slowed as it reaches the AVN, allowing time for the atria to contract and fluid to move prior to activation of the ventricular chambers of the heart. The impulse then travels through the His bundle, bundle branches, and the Purkinje system, enabling coordinated contraction of the large ventricular chambers of the heart.

Importantly, each subcomponent of the CCS is largely unique in both form and function from other regions of the heart. Anatomically, cells of the SAN and AVN are generally smaller than other myocardial populations, lack highly organized sarcomeres, and have relatively simple membrane structures (no intercalated disks or T-tubule systems) [1,2,3,4,5]. These cells are also coupled through relatively high resistance gap junctions [6,7,8,9] and rely heavily on calcium currents for the upstroke of their action potentials [10,11,12]. As a result of these anatomic and molecular characteristics, electrical propagation through SAN and AVN cells tends to be very slow.

Features present in nodal cells are quite different from the rapidly conducting components of the His–Purkinje system. While species differences exist, in general, Purkinje fiber cells are larger than the working ventricular myocardium [13], express elevated levels of the low-resistance gap junctional protein Cx40 [8,14], and utilize rapidly activating sodium currents for depolarization [15,16]. Consistent with nodel cells, however, His–Purkinje cells also tend to lack dense sarcomeres and display few T-tubules [17].

From a physiological standpoint, the most prominent feature of the nodal populations is the presence of slow diastolic depolarization (Figure 1B). During the heart’s resting phase, nodal cell membranes slowly depolarize as a collection of mutually entrained cell-surface ion channels, pumps, and sarcoplasmic reticulum-based proteins allow for cytoplasmic accumulation of positively charged ions. This serves to depolarize the cell membrane, stimulating voltage-dependent ion channels to open, which allows the initiation of an action potential [10,18,19,20]. Cells within the SAN have the fastest intrinsic rate of activity and therefore serve as the primary pacemaking region of the heart. His–Purkinje cells can be identified physiologically on the basis of parameters including a rapid upstroke velocity, a more negative resting membrane potential than the working myocardium, and an elongated action potential duration (Figure 1B) [10,21].

Interestingly, as the heart forms and becomes functional in the embryo, none of the cell types present within the mature CCS are present. Determining how the complex cellular circuitry that comprises the CCS is organized and integrated into the existing developmental framework of the heart has, therefore, remained one of the most fascinating and challenging topics associated with embryological cardiac morphogenesis. It has now become apparent that components of the conduction system become functional in a proximal-to-distal sequence, with the pacemaker cells of the SAN differentiating during heart looping stages [22], preferential conduction tracts through the atria forming during early septation stages [23,24], the AVN and His bundle emerging towards the end of ventricular septation [23,25], and the ventricular Purkinje fiber network maturing following the completion of cardiac morphogenesis [26,27,28,29]. As such, the CCS represents a biorhythmic cellular network that forms and becomes functional within a field of pre-existing electrically active working myocardial cells, meaning that each cell population within the conduction system must acquire specialized electrophysiological characteristics and establish the appropriate connections with the adjacent components of the network while the heart is actively beating. Given the complexity of cardiac morphogenesis, examining how these processes occur has, and continues, to require unique physiological tools. A major focus of this review is to introduce some of the imaging techniques that have helped to demonstrate the functional maturation of the developing heart, provide a brief historical perspective on the adaption of those techniques for embryonic cardiac physiology, and project how novel imaging and cell biological techniques may help to push the field forward in the coming years.

## 2. Historical Perspective

While our modern understanding of the cellular, molecular, and physiological events that dictate electrical patterning of the heart continues to reveal unexpected details, the underlying fascination with the rhythmic, coordinated beating of the developing heart extends back millennia. Much of this interest has stemmed from the simple satisfaction provided by watching the rhythmic cycle of contraction in the embryonic heart. Indeed, observational accounts of the developing avian heartbeat date as far back as Aristotle [30]. It is not surprising, therefore, that much of the research focused on trying to understand the nature and developmental emergence of cardiac biorythmicity has initially been based on visual observation. For instance, as early as 1890, Fano and Bandano constructed an ingenious method of projecting light though embryonic cardiac tissue onto moving film. In doing so, their studies produced the first recordings of embryonic heart activity. In this pioneering study, regional differences in the innate rate of activation between subdomains of the heart were first documented, the requirement of the atrioventricular junction for synchronization of the forming atria and ventricles was noted, and the speed of signal transmission in the early heart was first estimated at 6–8 cm/s (reviewed by [31,32,33]). The advent of a microscopy technique referred to as micromoving pictures by Patten [34] further pushed the boundaries of early developmental cardiac research. In 1920, Sabin published the first report capturing the timing and location of the initial contractions of the embryonic chick heart tube, and, expanding on this, Patten and Kramer were able to construct a detailed analysis of how the first contractions of the heart related to the morphogenetic fusion of the embryonic cardiac primordia [34]. Predictably, an array of film-based recording studies followed these early reports, as researchers began to recognize that visual recordings simultaneously provided large amounts of both spatial and temporal information regarding the activity of the heart, a concept that remains a principle component of the optical techniques used today for developmental cardiac physiology.

In parallel with these early visual-recording-based studies, the electrical nature of the developmental cardiac activity was first explored as electrocardiograph techniques were adopted for use on embryonic tissue. In 1913, Wertheim–Salomonson succeeded in detecting extracellular electrical fluctuations from chick embryos. While hampered by the sensitivity of the detection equipment used, these studies were able to demonstrate clear electrical deflections initiated in developing hearts [34]. With improvements in technology, in particular, the development of signal amplifiers, a cohort of investigators were able to record electrical activity from successively younger embryos spanning looping stages of cardiac morphogenesis through septation stages [35,36,37]. Perhaps most significantly, these investigators were able to demonstrate that features present in the adult echocardiogram (ECG) could be detected in hearts as young as late looping stages, confirming the multi-chambered activation sequence present in the adult heart (atria followed by ventricles) emerging during early embryonic development.

The ability to directly record changes in membrane potential via intracellular microelectrodes was first utilized in the developing heart in a series of studies conducted by Fingl, Woodbury, and Hecht. In these studies, the maturation of the electrical impulse shape over cardiac development was first noted, and how the developmental cardiac action potential responded to chemical treatment was explored [38]. In the 1960s, Van Mierop was able to expand on this work, becoming the first investigator to record action potentials from cardiomyocytes as early as heart tube stages, and noting that regional differences in action potential kinetics emerge during looping morphogenesis [39]. The subsequent progress of electrical recordings of developing cardiomyocytes has been extensively reviewed elsewhere [40]; however, the impact that these earliest studies have had on our fundamental understanding of the electrical maturation of the heart cannot be overstated.

**Figure 2:**
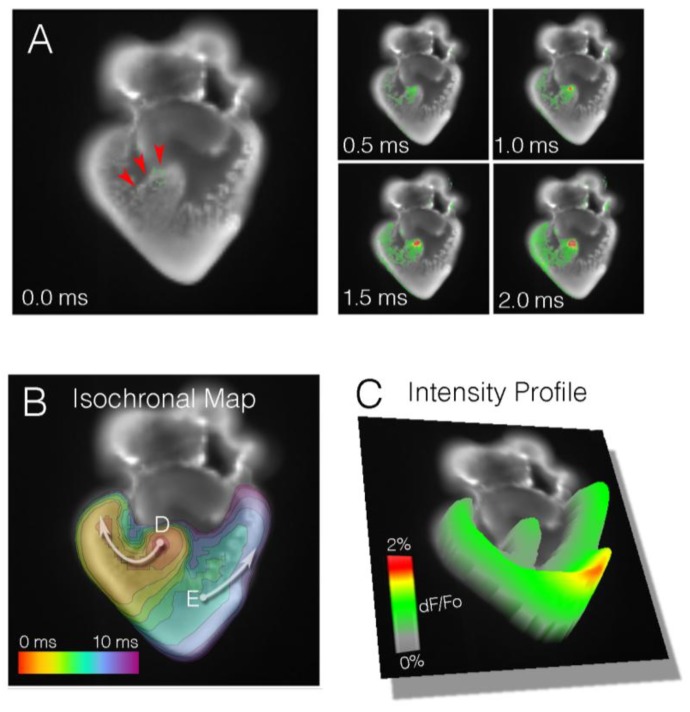

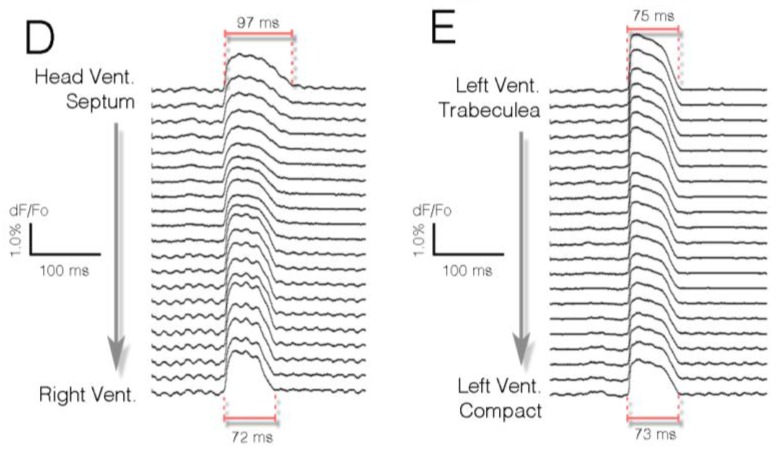
Optical mapping allows for integrative, simultaneous acquisition of physiological parameters from multiple points in situ. (**A**) Time series of electrical impulse propagation through a section of embryonic cardiac tissue. In this example, a Hamburger and Hamilton (HH) stage 30 [41] embryonic chick heart was sectioned in the coronal plane and live imaged at 2000 frames per second. Activation of the right bundle branch (red arrowheads) can be seen; (**B**) Isochronal map (1 ms/div) depicting the ventricular activation pattern of the heart from (**A**); (**C**) Intensity plot indicating relative signal amplitude (dF/Fo) for heart in (**A**); (**D**) Stripe analysis from region noted in (**B**). These data track the change in action potential shape as the impulse moves from the His bundle towards the right ventricle. Note that action potential duration decreases; (**E**) As in (**D**), tracing of action potential characteristics as it propagates from left ventricular trabeculae to compact myocardium.

Throughout the first half of the 20th century, both visual recording and electrical sampling were used independently to trace how morphogenesis and physiology cooperatively evolve as the heart forms. However, it did not escape investigators’ attention that technical limitations with both techniques challenged the ability to develop a comprehensive model of cardiac functional maturation. While video recordings provided an overview of organ-level activation and could be used to construct longitudinal studies of cardiac development, data was largely based on the physical movement of the tissue. As this is an indirect measure of physiological behavior, the underlying regional differences present among various populations of cardiomyocytes could not be directly assessed. In contrast, electrical recordings provided detailed information regarding the kinetics of membrane dynamics in various regions of the heart, but these studies were technically challenging and provided only limited spatial information, making quantification of parameters including the directionality of impulse propagation and the conduction velocity relatively inaccessible. In addition, the inability to impale small cells (<3–5 µm in diameter) with microelectrodes and the fact that contractile movement frequently disrupts the membrane seals required for stable recording represent significant challenges for conducting such developmental cardiac electrophysiology studies. Therefore, it is not surprising that a merger of optical recording and electrical sampling emerged as a method to circumvent the limitations of these techniques, as being able to directly visualize electrical activity simultaneously from many sites in a tissue preparation without physically disrupting the sample combined the most attractive components of each technique. As summarized in Figure 2, the ability to quantitatively examine electrical activity across a whole tissue can be used to examine a variety of physiological parameters in ways that would be exceedingly challenging using other methodologies.

## 3. Optical Mapping for Developmental Cardiac Studies

The push to develop optical techniques for monitoring electrical activity was most prominently driven by researchers in neuroscience. The vast majority of what is known regarding the voltage-dependent membrane currents that drive changes in membrane permeability comes from either intracellular or patch clamp measurements of membrane potentials. While extraordinarily powerful, these techniques require a large degree of expertise and have a few critical limitations. From the perspective of a neuroscientist, microelectrode-based techniques are not able to monitor behavior at multiple sites along a cell; nor are they fine cytoarchitectural features accessible for recording (i.e., small dendritic or axonal projections). As such, starting in the 1960s, optical techniques for examining changes in membrane potential began to be explored, with the underlying rationale being that visual recording of electrical activation could be used to directly evaluate changes in membrane potential from multiple regions of cells/tissue without the risk of physical damage. The list of techniques, reagents, and indicators that have been developed for such purposes is quite expansive [42], and, given the focus of this review, we have chosen to highlight the evolution of reagents that have been widely used in the developing heart.

### 3.1. Voltage-Sensitive Dyes

Initial endeavors at tracking electrical activity with optical instrumentation were based on birefringence principles [43,44]. The theory behind this was that changes in electrical field intensity across a membrane influence the polarization or light-scattering properties of that membrane, which could be projected to a detector such as a photomultiplier. While such techniques are still utilized [45], they suffer from poor signal amplitude and a low signal-to-noise ratio. As a result, attempts were made to screen fluorescent dyes for spectral shifts that correlated with changes in membrane potential [46,47,48]. Most membrane depolarization events in adult excitable cells have a magnitude of approximately 100 mV. While this is relatively small when compared to most of the electronic systems we routinely interact with, when considerations for membrane geometry are made, millivolt differences across a cell membrane can support voltage gradients greater than 10^5^ V/cm^2^ [49]. Operating under the hypothesis that electrical fields of this size should be sufficient to alter the optical absorption/emission characteristics of chromophores, thousands of dyes were initially evaluated for potentiometric activity. This led to the initial identification of merocynanine dyes as having characteristics sufficient for voltage imaging [46,47,48]. These dyes responded to voltage changes with a linear shift in fluorescence intensity, displayed rapid spectral shifts as cells depolarized, and had adequate signal-to-noise ratios to allow for direct monitoring without substantial signal averaging. 

Following the initial identification of the merocynanine dyes, Leslie Loew’s laboratory conducted a series of rational design studies in an effort to further improve and optimize dyes for live imaging of voltage changes in excitable tissues [50,51]. These studies yielded many of the voltage-sensitive dyes (VSDs) that have been and are still widely used for optical imaging of voltage changes [52]. Among these, Di-4-anepps has been, perhaps, the most popular potentiometric dye used for embryonic heart studies. 

**Figure 3:**
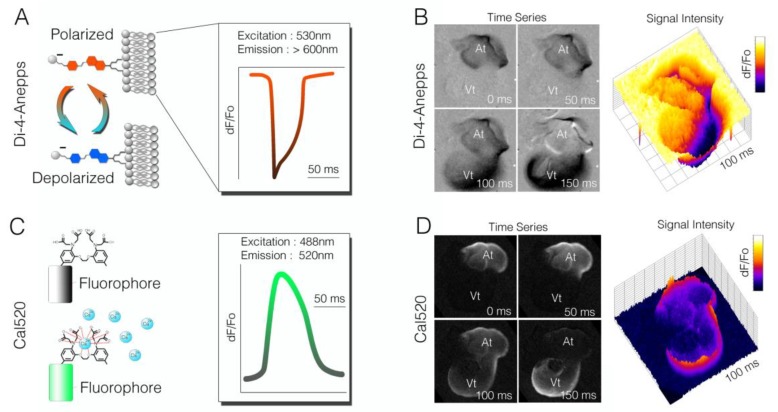
Comparison of voltage-sensitive dyes to small-molecule calcium indicators. (**A**) Diagram indicating change in spectral characteristics for the voltage-sensitive dye, Di-4-anepps. Depolarization causes a blue shift in the dye. In this example, an imaging system was designed to image long wavelengths (red), and a drop in fluorescence intensity correlated to an action potential being fired; (**B**) Time series of raw imaging data acquired from a Hamburger and Hamilton (HH) stage 18 chick heart stained with D-4-annepps. The drop in intensity was seen as the cardiac action potential propagated from atria to ventricle; (**C**) Diagram of the small-molecule calcium indicator Cal520. Calcium ion occupancy in the ETGA/BAPTA-like motif of the molecule increased fluorescence output, which could be imaged with most standard eGFP filter sets; (**D**) Raw imaging of Cal50 dF/Fo in a HH stage heart. Unlike Di-4-anepps (**B**), Cla520 increased its fluorescence signal upon depolarization.

Di-4-anepps was engineered with several features that make it well suited for developmental cardiac imaging. Di-4-anepps belongs to a class of Styryl dyes that show rapid response and a relatively large change in fluorescence per millivolt (mV)of depolarization [53]. The amplitude of the Di-4-anepps response is particularly useful in preparations of embryonic cardiac tissue, for which cells achieve only a fraction of the membrane polarization seen in adult cardiomyocyte [54]. In general, the structure of Di-4-anepps can be subdivided into three functional regions: a hydrophobic group for membrane anchoring, a voltage-sensitive chromophore, and a charged moiety to orient the dye molecule perpendicular to the cell membrane and slow internalization. The chromophore can be further subdivided into an electron-rich pi system, linker, and electron-deficient pi system. Collectively, these features allow the dye to embed into the outer leaflet of the plasma membrane (via a pair of hydrophilic hydrocarbon chains) in an orientation parallel to the electrical field generated by the cell’s membrane potential. Excitation of the dye shifts the electron configuration in a manner that is sensitive to the local electrical field, resulting in membrane-potential-dependent alterations in emission wavelength [50] (Figure 3A). As a cell depolarizes, the dye shifts its emission spectra, which can be detected as a decrease in intensity in longer (red) wavelengths in favor of increased intensity in shorter (blue) wavelengths. This change in fluorescence (dF/Fo) is linearly associated with changes in membrane potential and can be used to monitor and trace waves of depolarization (Figure 3B). Instrumentation is, therefore, designed to capture these rapid changes in fluorescence intensity. While Di-4-anepps is widely used, some of its limitations should be noted. Di-4-anepps has been shown to alter action potential kinetics (particularly conduction velocity and action potential duration) with prolonged imaging [55,56], and, as with all potentiometric dyes, Di-4-anepps does not provide an absolute value for membrane potential, only a relative change in fluorescence.

### 3.2. Calcium Dyes

As with VSDs, calcium indicators have been broadly applied to both neural and cardiac imaging modalities to trace cellular excitability. Membrane depolarization in cardiomyocytes leads to the opening of cell-surface voltage-dependent, calcium-selective ion channels. This results in calcium ion influx, which triggers a release of calcium from intracellular sarcoplasmic reticulum stores. Calcium-induced calcium release drives cytosolic calcium to increase from nanomolar up to millimolar concentrations, triggering sarcomere contraction. Therefore, monitoring changes in the cytosolic calcium concentration is an effective, albeit indirect, method of visualizing cardiac electrical propagation. Several classes of calcium dyes have been generated over the years, with those most prevalently used including the Indo, Quin, Fura, and Fluo dyes. Each have undergone multiple rounds of iteration to optimize fluorescence, permeability, toxicity, and kinetics, and dozens of indicators are now commercially available [57]. These indicators all share an octacoordinate binding site based on the structure of the calcium selective chelator EGTA [58,59]. When occupied by a calcium ion, this region of the dye molecule elicits structural changes in the attached fluorophore, leading to calcium-dependent increases in fluorescence (Figure 3C). While these indicators have slower on/off kinetics than potentiometric dyes such as Di-4-anepps, the change in fluorescence upon electrical stimulation are orders of magnitude higher than VSDs. This can be of great utility for measuring activity from immature cardiomyocytes, for which shallow polarization (and thus low action potential magnitude) can limit the effectiveness of VSDs. Just as with VSDs, considerations should be made for the structure and nature of how these indicators work when selecting them for use. One primary concern when using calcium indicators is choosing a compound with the appropriate affinity. High-affinity dyes can act as intracellular calcium buffers, actively interfering with normal cycling dynamics and distorting measurements. Therefore, the most accurate measurement of calcium dynamics is in fact achieved by using an indicator with the lowest calcium affinity that can be practically imaged. This often requires testing a few reagents across the spectrum of potential dyes. For instance, we have tested several calcium dyes with developing avian cardiac tissues, including Fura-2 (Kd ~ 140 nm), Rhod-2 (Kd ~ 570 nM), and Flou5F (Kd ~ 2.3 uM), and found that Cal520 (Kd ~ 235 nM) provides effective signal-to-noise and strong intensity shifts when imaged from heart tube to looping stages (Figure 3D).

### 3.3. Genetically Encoded Indicators

While both potentiometric and ion-sensitive dyes have been used with great success for tracking physiological dynamics in the developing heart, improvements in genetically encoded indicators (GEIs) for voltage and calcium have greatly broadened the available tool kit for cardiac imaging. As with the dye-based approaches discussed above, GEIs have gone through multiple rounds of design-based iteration in order to achieve sensitivity, dynamic ranges, and signal-to-noise ratios comparable to small-molecule indicators. A large array of GEIs now exist, with differing spectral characteristics, affinities, and intensities [60,61]. Among the most appealing aspects of the GEIs is that they do not require a staining procedure, which can be difficult to target to specific cell types. Cell-type-specific promoters can be utilized to drive the expression of GEIs in specific domains in the embryo, and, unlike the dye-based strategies, physiological behaviors can be monitored over prolonged periods. Furthermore, GEIs are being increasingly used to perform high-throughput evaluation of functional characteristics in stem-cell or somatic-cell reprogramming techniques for generating cardiomyocytes [61,62,63,64,65,66], in which staining procedures and dye toxicity are major limitations. Thus far, the most widely used GEIs for calcium imaging in the developing heart are based on GCaMP. GCaMP was engineered to contain a circularly permuted eGFP flanked by the M13 fragment of myosin light-chain kinase on one side and calmodulin on the other. In the presence of calcium ions, a calcium–camodulin-M13 interaction is stabilized, resulting in a conformational change in the eGFP that increases its fluorescence output [67]. GCaMP2 was the first genetically encoded calcium indicator to be used to evaluate calcium transient behavior in a developing four-chambered heart [68], and successive improvements on the basic structure of GCaMP have led to its use in a variety of developmental model organisms.

While the panel of genetically encoded voltage sensors for optical recording is expanding at a rapid rate [69], their use in developmental cardiac studies remains far less prevalent than calcium indicators. As these reagents improve, however, it is likely that their inclusion in developmental studies will become more common.

### 3.4. Basic Instrumentation

Instrumentation for physiological imaging of the developmental heart does require some specialization to accommodate challenges that are not necessarily as significant in adult tissue. As mentioned above, developing cardiomyocytes are not as polarized as their adult counterparts [54], particularly before ventricular septation has completed. This effectively means the amplitude of the action potentials that juvenile cardiomyocytes exhibit can be quite small and, as a result, changes in fluorescent indicator intensities are not as pronounced as in adult tissue. Furthermore, the developing heart is, of course, far smaller than in the adult. The fewer cells and thinner wall thickness present during development mean that the volume of excitable cells from which a signal can be recorded is far less than in the adult. As such, imaging systems for developmental studies must be optimized for maximal light-collecting ability.

Currently there are a multitude of imaging components that can be combined to construct a basic microscopy system for optical mapping purposes. In general, the major considerations in designing these systems are related to speed, magnification, and sensitivity of light detection.

A stable, high-intensity light source is preferable for any optical mapping system. The mercury arc light sources used for most fluorescent imaging techniques are robust and bright and have a large array of flexible uses, but tend to show too much fluctuation in output intensity over the millisecond time scales in which physiological imaging studies are conducted. Tungsten halogen lamps display improved stability over mercury arc systems; however, halogen bulbs that can generate sufficient intensity have relatively short operational lives (approx. 100 h). As such, laser systems and light-emitting diodes (LEDs) are now the most common forms of illumination.

Most developmental imaging systems require at least some marginal magnification capacity, although this can vary greatly depending on the model organism (e.g., a fully looped zebrafish heart is approximately 200 µm in length, whereas a fully septated chick heart is approximately 1 cm in length). In general, the objective characteristics most ideal for developmental optical mapping are a long working distance and high numerical aperture. Typically, trade-offs must be made between these two parameters to meet the needs of the sample to be examined.

As with objectives, similar trade-offs must also be made when selecting detectors for optical mapping. Generally, this trade-off manifests in sacrificing speed and sensitivity for the number of pixels that can be simultaneously imaged or vice versa. For example, the upstroke component of a cardiac action potential is in the order of 2 ms [70]. In order to acquire multiple time points during the upstroke event, imaging may need to be conducted at 1–2 kHz. Very few camera systems with megapixel densities are capable of imaging at that speed while also being able to detect small deviations in fluorescence intensity. Historically, photodiode arrays were the first detectors used for optical mapping in cardiac tissues. These were fairly low resolution systems consisting of only a few sampling regions [32]. However, the photodiode arrays provided a high dynamic range, high acquisitions speeds, and good signal-to-noise. Progressively, advancements in CCD and CMOS camera chip technologies have supplanted the earlier photodiode arrays. The major advantages with CCD and CMOS cameras were the increased pixel density they could achieve and the relative ease of use. The last decade has seen an uptick in cameras manufactured expressly for optical mapping techniques, with notable companies including RedShirt Imaging (Decatur, GA, USA) and SciMedia (Costa Mesa, CA, USA), who each offer a variety of camera systems with differing temporal and spatial specifications. However, when imaging for calcium transient behavior (typically on the order of 50–100 frames per second), more standard, commercially available cameras, including those from Hamamatsu (Bridgewater, NJ, USA) and Andor (Belfast, UK), which have a higher spatial resolution, are often adequate.

In summary, reagents and equipment for conducting a variety of imaging-based investigations into the electrical maturation of the heart are now widely available. The diversity and flexibility of these tools is opening opportunities to investigate developmental cardiac physiology from excitingly novel perspectives. As described below, effective application of these techniques has already provided fundamental insight into the structure–function dynamics of cardiac morphogenesis.

## 4. Application of Physiological Imaging to Understand Cardiac Electrophysiological Maturation

Through a series of imaging studies spanning the last 40 years, a wealth of detail has emerged regarding how developmental transitions in cardiac morphogenesis correlate to changes in electrophysiological properties. Pioneering work in this field was initiated by Kohtaro Kamino’s group, who began using potentiometric dyes to evaluate electrical activity in cardiac progenitors as well as in the early heart tube [32,71,72,73]. Given the limitations of intracellular recording techniques noted above, these studies were among the first to examine electrical activation and propagation at these early stages of cardiac development. Strikingly, through a series of reports, the imaging system designed by the Kamino lab was able to identify electrical activity in the early lateral plate mesoderm, prior to the initial fusion of the progenitor cells that make up the heart tube, well before the onset of cardiac contraction [32,71,72]. Initially, electrical activity was restricted to a small region of the mesoderm, and the amplitude of the recorded signals was quite small [32]. However, over successive stages, the region of the heart primordia that appeared to be spontaneously excitable expanded, resulting in propagating waves that moved through the pre-contractile myocardium. Recent studies using the calcium indicator Cal520 have confirmed that propagating transients are present during cardiac crescent stages in the mouse embryo, consistent with the studies conducted in the chick almost 40 years ago [74].

**Figure 4:**
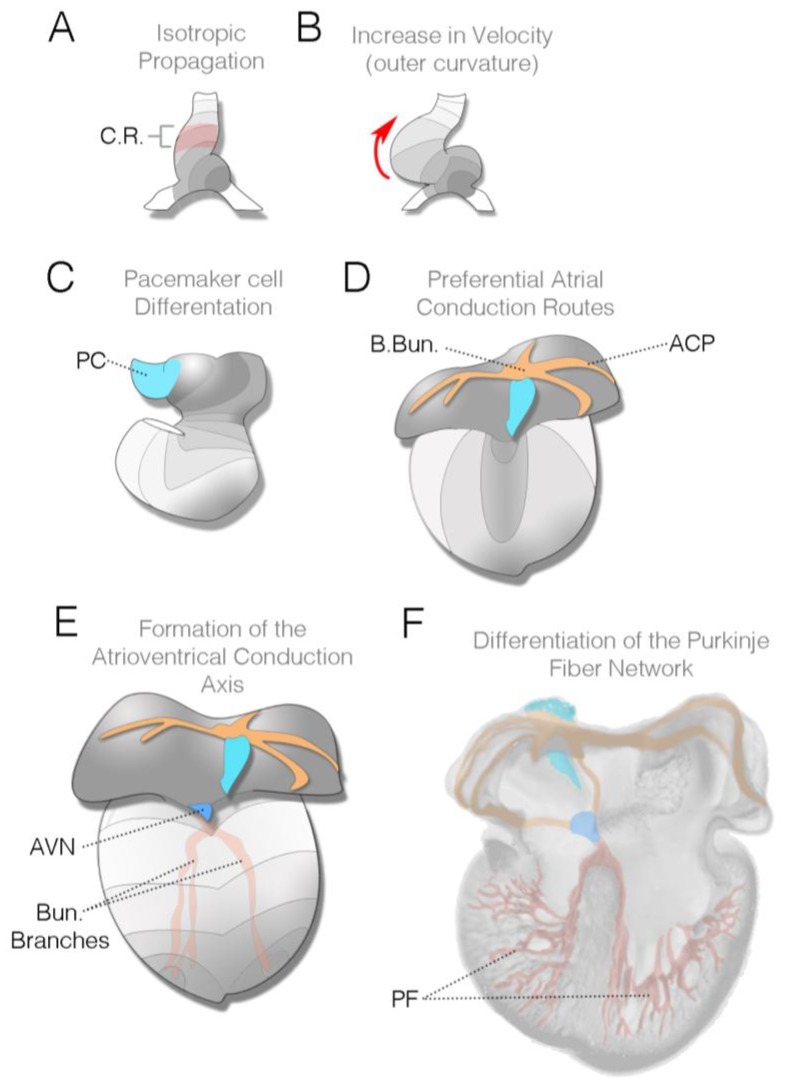
Changes in conduction patterning during cardiac morphogenesis: (**A**) Diagram of heart tube viewed from the ventral surface (Hamburger and Hamilton (HH) stage 10 in chick, E8.25 in mouse). This stage corresponds to the first observable contractions in the heart; contractile region (C.R.) is noted in red. Electrical impulses initiate in the left inflow region of the heart (grey region) and propagate as a uniform wave towards the outflow; (**B**) As cardiac looping progresses (HH stage 15 in chick, E8.5 in mouse), the impulse increases velocity along the outer curvature of the heart (red arrow); (**C**) Towards the end of looping, cardiac pacemaker cells (PC) differentiate and are added to the heart (light-blue region). These cells will make up the muscular component of the mature sinoatrial node (HH stage 18 in chick, E9.5 in mouse); (**D**) Diagram of an early septation stage heart viewed from the dorsal surface (HH stage 23–25 in chick, E10.5–E12.5 in mouse). During these stages, atrial muscle bundles, including Bachmann’s bundle, elaborate and establish themselves as atrial conduction pathways (ACP—orange); (**E**) As ventricular septation completes, the atrioventricular conduction complex, including the atrioventricular node (AVN; dark blue), His bundle, and bundle branches (red) become functional HH stage 25–30 in chick. As noted in the text and in Figure 2, the right bundle branch frequently becomes active prior to the left bundle branch; (**F**) Dorsal half of the heart viewed from the front. After the termination of cardiac morphogenesis, the distal Purkinje fiber (PF) network differentiates in subepicardial and/or perivascular regions in the heart. All diagrams are based on chick cardiac development.

As the heart tube initially forms, the electrically active area is larger than the contractile mass [22,39,75]. At these stages, impulses initiate in the left posterior inflow segment of the heart tube and propagate as a uniform wave toward the most anterior segment of the heart [22,39,73], whereas only a small region of the more anterior myocardium displays contractile movement [31] (Figure 4A). Parameters of the electrical impulse are quite immature at these stages, displaying relatively slow upstroke velocities and elongated action potential durations [39,75,76]. Furthermore, the frequency of activation is also slow, as the cycle length between successive electrical impulses can exceed 1–2 s.

Regional differences in conduction characteristics emerge as the heart undergoes cardiac looping. The conduction velocity begins to increase along the outer curvature of the forming ventricle as the heart begins to loop [22,77,78] (Figure 4B), and, coincidently with the emergence of the endocardial cardiac cushions, a region of slow conduction emerges at the atrioventricular junction separating the embryonic atrial and ventricular chambers [39,76]. This marks the embryonic equivalent of the slow conducting AVN present in the mature heart. The activation pattern of the heart at this stage of development remains unidirectional, initiating in the inflow region of the heart, propagating across the primitive atria, and then proceeding from the base of the ventricle towards the cardiac outflow.

Towards the end of cardiac looping, pacemaker cells emerge within the right inflow component of the heart [22]. Unlike the atrial and ventricular primordium that are added to the heart at earlier developmental stages and appear to transition through intermediate electrically active states, bona fide pacemaker cells show functional characteristics far closer to their adult counterparts when they first differentiate. Optical recordings demonstrate that these cells display a pronounced slow diastolic depolarization and a much higher frequency of depolarization than either atrial or ventricular progenitors [22]. Around the same time that pacemaker cells are differentiating, subtle changes to ventricular activation patterns are also occurring. Imaging from chick, mouse, and rat embryos demonstrates that the conduction pattern present in the ventricles begins to evolve as a propagation route emerges along the ventral anterior surface of the myocardium, roughly corresponding to where the ventricular septum will form [77,79,80]. Interestingly, this pattern emerges prior to the completion of ventricular septation and precedes the formation of the His bundle and bundle branches.

As looping terminates, the atrial conduction pattern begins to transition. Initially, conduction across the atria proceeds as an even isotropic wave moving from the pacemaking region to the atrioventricular junction; however, as the pectinate muscle bundles of the atria form and enlarge, these structures become preferential conduction conduits through the atrial myocardium [23,24] (Figure 4D). A large bundle along the roof of the atria, known as Bachmann’s bundle, becomes one of the principle routes by which impulses generated in the SAN move from the right atria to the left [23]. More generally, however, as pacemaker-initiated action potentials enter the roof of the atria, the large pectinate muscles coordinate atrial activation by rapidly conducting impulses towards the forming atrioventricular conduction axis. While the atrial muscle bundles do not meet the formal anatomical definition of a specialized conduction network [3], recent reports have indicated that they are enriched with several factors associated with a rapid conduction velocity, including CX40 and SCN5a [24,81].

Slow conduction through the entire atrioventricular myocardium persists through the completion of cardiac looping and is eventually terminated by the formation of an insulating fibrous ring between the atria and ventricles. This prevents electrical communication between the upper and lower chambers of the heart, except through the forming atrioventricular conduction axis. As the ventricular septum partitions the right and left ventricles, conduction through the bundle branches can be detected (Figure 2 and Figure 4E). Optical mapping studies performed on a variety of vertebrate models have demonstrated that the right bundle branch appears to become functional prior to the left bundle branch [79,80] (Figure 2). This formally marks the shift in the ventricular activation pattern from what is known as base-to-apex, to apex-to-base. The functional activation of the bundle branches precedes the establishment of the definitive ventricular fast-conducting conduction network consisting of Purkinje fibers. The Purkinje fiber network is the last component of the heart’s conduction system to terminally differentiate, becoming active during late gestational periods in avians and after birth in mice [26,29].

In general, studies focused on directly observing transitions in the heart’s conduction patterns as morphogenesis progresses have established that drastic changes in activations patterns occur during cardiac development. Currently, the vast majority of these studies have been limited to imaging embryos using two-dimensional modalities. Moving forward, adaptive technologies that can integrate multidimensional imaging over several developmental windows will be required to push our understanding of how the electrical syncytium of the heart patterns. Several recent studies have fairly nicely demonstrated how the basic principles of physiological imaging outlined above are currently being supplemented by modern optical technologies to create new insights into cardiac electrophysiological maturation.

## 5. Recent Advancements

The ability to visualize impulse propagation across whole tissues has been critical for determining the stages at which cardiac conduction characteristics change and mature towards adult physiology. However, because of limitations born from the requirement of maximal light collection and high temporal resolution, much of the data collected to date regarding developmental cardiac physiology has not been multidimensional. This is by no means a critique of previous data or methodology, but the limitations of extrapolating what are essentially two-dimensional imaging series onto the true three-dimensional (3D) structure of the heart can lead to errors in calculating functional characteristics. For instance, it is difficult to account for electrical propagation pathways moving toward or away from a detector when considering absolute conduction velocity, and out of focus cells can complicate the interpretation of even ideal conduction pathways that are traversing perpendicular to the detector.

With this in mind, several studies have begun to push the imaging systems for optical physiology in the heart towards multidimensional interfaces. In a few such studies, a custom-built optical mapping system with adequate magnification specifications to image develop quail cardiac tissue was integrated onto an optical coherence tomography (OCT) platform. OCT is a non-invasive imaging modality based on measuring backscattered light intensity using low-coherence interferometry [82]. It does not require the introduction of contrast reagents and is non-destructive. Given its relatively high spatial resolution (2–20 µm), good penetration depths (1–3 mm), and high sampling rates (47 kHz), it can be used to rapidly construct 3D profiles of tissue architecture. Combining OCT with optical mapping allowed for data collected by imaging VSD responses to be mapped onto a 3D surface rendering of a mid-looping-stage quail heart. This allowed for the correction of conduction velocity speeds across the 3D architecture of the heart [78]. Subsequent studies using a combined OCT/OM imaging system have demonstrated that computational processing based on least squares optimization can further refine the 3D extrapolation of the conduction velocity in developing cardiac tissue [83].

Efforts have also been made to directly acquire 3D physiological data from embryonic cardiac tissue. Using a custom-built light-sheet microscope, Ma et al. were able to scan through a developing quail heart and computationally reconstruct an averaged electrical impulse propagation pathway to generate complete four-dimensional, volumetric recordings of cardiac activation maps [84].

Similarly, using the zebrafish heart, Weber et al. developed a light-sheet imaging system capable of creating high-speed imaging of calcium transient behavior across development [85]. This report demonstrated a scalable imaging system that can be used to quantify organ-level physiological dynamics but that also possesses sufficient resolution to quantify individual cellular behavior at multiple stages of cardiac morphogenesis.

The ability to stimulate and simultaneously record cardiac activity using a non-invasive, non-genetic, optical approach was recently explored by Wang et al. [86]. Using pulses of infrared point stimulation, the authors were able to effectively capture the heart rate without having to apply and an electrode-based stimulator.

The convergence of advanced fluorescent probes, genetic indicators, and imaging platforms is increasingly removing barriers towards both optically based recordings and light-induced manipulation of developing cardiac tissue. As such, it is not difficult to project the biological applications that studies in the near future will push these technologies towards.

## 6. Future Directions

Moving forward, a major challenge for developmental cardiac physiological imaging will be to develop methods that will allow for single-cell or subcellular resolution of functional dynamics in the forming heart. As current stem-cell and tissue engineering strategies for generating cardiomyocytes continue to advance, detailed understanding of the in vivo cellular and physiological maturation of cardiomyocytes will become increasingly relevant for the development of novel cellular-based therapeutics. A major technical barrier towards pushing stem-cell-based or regenerative medicine application towards clinical use is the relatively juvenile nature of cardiomyocytes that are generated through these techniques [86,87,88,89]. In this respect, direct physiological examination and manipulation at single-cell or subcellular resolution will provide critical insight for optimizing approaches for generating such cells ex vivo.

In addition, congenital heart defects (CHDs) represent the leading class of birth defects seen in humans. Correspondingly, considerable effort has been dedicated to understanding the molecular genetics of CHDs; however, how mutations in various pathways manifest at the level of the individual cellular function remains poorly understood. The major platforms available for studying genotype/phenotype relationships in developing cardiomyocytes have been dependent on the generation of transgenic model organisms or, more recently, the differentiation of patient-derived induced pluripotent stem cells [90,91]. While transgenic animal models represent the most powerful systems currently available for CHD research, it can be difficult to tease apart cell autonomous effects that result from genetic manipulation from those that are secondary to organ-level dysfunction. As such, iPSC systems represent a strong alternative approach; however, iPSC-derived cardiomyocytes are difficult to mature and lack the biomechanical environmental cues and electrical circuitry present in the heart [92]. 

**Figure 5:**
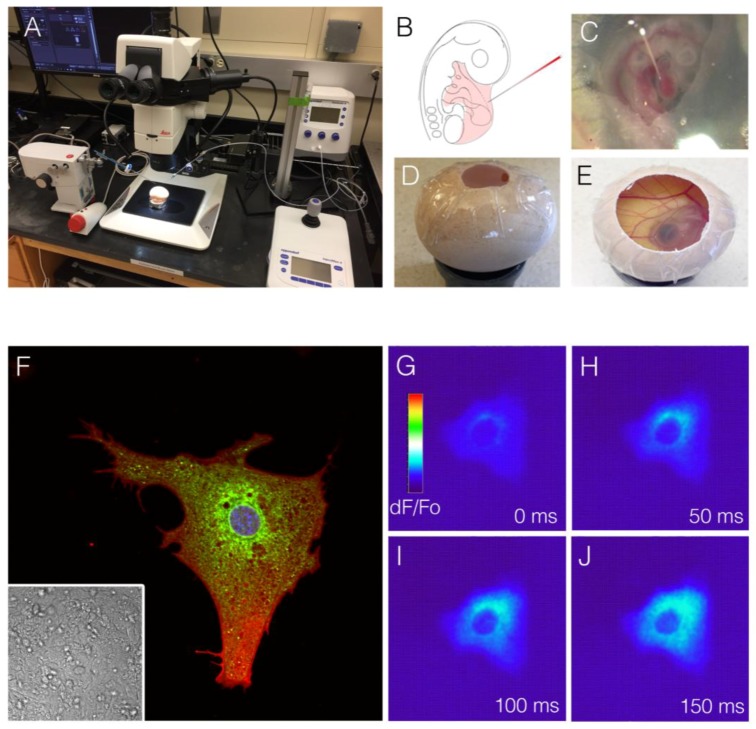
Mosaic analysis for imaging cardiac cell physiological maturation. (**A**) Microinjection system for viral- or plasmid-based transduction of cardiac cells; (**B**) Diagram of microinjection into the pericardial space of a chick embryo; (**C**) Photograph of injection procedure from (**B**); (**D**,**E**) Sealing and incubation of microinjected embryo to desired stage; (**F**) Embryonic cardiomyocyte transfected in vivo with a construct containing a membrane-targeted RFP, a sarcoplasmic-reticulum-targeted eGFP, and a nuclear-targeted BFP. This cell exists within a field of untransfected cardiomyocytes, as indicated by the phase contrast image (inset); (**G**–**J**) Time series of a cardiomyocyte from a similar stage as that depicted in (**F**), transfected with a construct encoding for the calcium indicator GCaMP6F. Cell was imaged at 100 frames per second. Calcium transients can be seen initiating in a perinuclear region (similar to the dense area of sarcoplasmic reticulum from (**F**)) and propagating out towards the cell periphery.

An alternative system to complement current transgenic animal-based manipulations may, therefore, be one that utilizes genetic and somatic transgenic approaches to generate mosaic cardiac tissues. The rationale behind such studies would be to engineer systems in which only small numbers of cells within the developing heart are manipulated, leaving the bulk of cardiac tissue in a wild-type configuration. Such approaches have several potential advantages. From the standpoint of physiological imaging, genetically encoded biosensors can be expressed in subpopulations of cells. The cells that carry the optically detectable biosensor can be easily identified while remaining directly in contact with their neighbors, and the behavior of the individual or small groups of cells can be assayed while maintaining the electromechanical syncytium of the heart. This allows for optimal imaging in the developing heart as single cells can be resolved against a background of negative cells (Figure 5). Unlike full transgenic animals, for which the vast majority of cells are labeled, such a mosaic system would allow for fine subcellular events to be live imaged in vivo. While not yet broadly utilized in developmental cardiac research, inspiration for this type of approach can again be drawn from neuroscience research. Particularly in regard to physiological imaging, several strategies for creating mosaic neural networks, in which subpopulations of cells are labeled and examined, have been used with great success.

A classical approach toward understanding single-cell functional dynamics within the brain involves delivering small-molecule dyes specifically to cells of interest via a patch-clamp pipette. While effective for local delivery, this approach can be technically challenging, and the calcium/voltage reporters used for such studies are typically not amenable to long-term imaging, as their phototoxicity precludes longitudinal studies. Furthermore, it can be challenging to target such dyes to specific cell types during microinjection. The use of GEIs, however, has effectively been applied to circumvent these issues. Approaches including viral gene delivery and in vivo electroporation have been utilized to directly introduce molecular tools for optical physiology in the brains of wild-type or genetically modified animals [93,94]. Both of these systems can take advantage of localized targeting; cell-type-specific promoter designs; Cre/FLP recombinases; and, in the case of viruses, differential infectivity to generate the level and specificity of integration required for the desired experimental conditions.

Viral-based somatic transgenesis has been used in cardiac research for over 20 years [95,96,97,98]. Retroviral-based genetic lineage tracing and overexpression studies are commonly performed in avian embryos, and several recent studies in rodents have demonstrated the utility of adeno-associated virus (AAV) for the in vivo generation of mosaic cardiac tissue [99,100]. Therefore, viral-mediated somatic transgenesis is an attractive approach for generating myocardial populations for in situ single-cell live imaging. In addition, while electroporation is difficult to perform in the developing heart, recent studies have demonstrated that various chemical-based transfection techniques are possible during early avian cardiac morphogenesis [101,102]. Indeed, our group has conducted initial proof-of-concept studies in the chick embryo, confirming that transfection-based introduction of the calcium indicator GCaMP6F can be used to generate cardiac mosaics in which calcium transient behavior can be imaged across different stages of cardiac development at the subcellular resolution (Figure 5). Moving forward, it is not difficult to foresee experimental approaches in which disease-relevant pathways are disrupted in cells co-transfected with physiological indicators, allowing for the functional consequences of genetic perturbations to be read out in real-time. Furthermore, given the success of optogenetic approaches in the adult heart and developing heart [103,104,105,106], somatic transgenic approaches open an exciting avenue towards conducting light-based manipulations of both physiological and biochemical processes in developing cardiomyocytes in vivo with a high degree of targeting specificity.

In summary, the imaging and molecular biology approaches that are coming online across the spectrum of in vivo and in vitro biomedical research are drastically altering the way cellular physiology is interrogated. As developmental cardiac research continues to elaborate and construct tractable means of porting these techniques into the embryonic heart, the technical hurdles towards the systematic, scalable investigation of cardiac physiological maturation are becoming less prohibitive, opening exciting and novel means to investigate the mechanisms of cardiac functional maturation.
